# Primary epidural lymphocyte-depleted Hodgkin’s lymphoma of the thoracic spine – presentation of a rare disease variant

**DOI:** 10.1186/1471-2377-12-64

**Published:** 2012-08-03

**Authors:** Ekkehard M Kasper, Fred C Lam, Markus M Luedi, Pascal O Zinn, German A Pihan

**Affiliations:** 1 Division of Neurosurgery, Beth Israel Deaconess Medical Center, Harvard Medical School, Boston, MA, USA; 2 Department of Pathology, Beth Israel Deaconess Medical Center, Harvard Medical School, Boston, MA, USA; 3 110 Francis Street, Suite 3B, Boston, MA, 02215, USA

**Keywords:** Spinal cord compression, Primary, Lymphocyte-depleted Hodgkin’s lymphoma

## Abstract

**Background:**

Lymphocyte-depleted Hodgkin’s lymphoma is the rarest form of classical Hodgkin’s lymphoma, accounting for < 1% of all cases. Patients often have advanced-stage disease at the time of presentation with an aggressive clinical course. Even more uncommon is primary extranodal disease and rarely it will be presenting with spinal cord compression.

**Case presentation:**

An 88-year-old Caucasian female presented with a history of upper back pain for several months and new onset bilateral leg numbness and weakness. MRI of the spine showed a dorsal epidural lesion with cord compression at T1-T4 with involvement of the paraspinal muscles. The patient received urgent surgical decompression, with final histopathology showing a lymphocyte-depleted Hodgkin’s lymphoma. Systemic work-up did not show evidence of nodal disease. Following surgery, she received a course of radiotherapy with good outcome.

**Conclusion:**

To the best of our knowledge, this is the first reported case of primary lymphocyte-depleted Hodgkin lymphoma presenting as epidural spinal cord compression. Our report, in conjunction with a review of the literature, suggests that surgical intervention is clearly indicated in *de novo* disease followed by radiotherapy.

## Background

Hodgkin’s lymphoma (HL) usually presents with painless enlargement of peripheral lymph nodes, mostly in the cervical region in 90% of cases, with only 10% arising from extranodal regions [1]. Approximately 5-20% of patients with HL develop osseous involvement during the course of their disease [2-6], but only 0.25% present in a primary fashion [7].

Epidural lymphomas are observed in 0.1-6.5% of all lymphomas and only 5% of patients with HL develop spinal cord compression during the course of their disease [8-10]. There are only a handful of reports of primary epidural spinal HL (PESL; Table 1) [1,8,9,11-13][14,15].

**Table 1:** Evidentiary table of case reports of primary epidural spinal Hodgkin’s lymphomas

**Case Report**	**Patient**	**Levels of Disease**	**Neurologic Findings**
Al-Khayat H *et al,* 2007	47 yo female	C7, T1	Bilateral hand weakness
Cagavi F *et al*, 2006	30 yo male	C6, L3	Low back pain
Citow JS *et al*, 2001	54 yo female	T4,5	Upper back pain
Higgins SA *et al,* 1995	46 yo female	C5-T8	Lower extremity numbness
Illes A *et al,* 2002	32 yo male	T11-12	Paralysis

Patients usually present with back pain and/or neurologic deficits specific to the level of the lesion, along with lymphoma-related B-type symptoms of weight loss, night sweats, and fever. Tumors have a preference for the dorsal aspect of the thoracic spine, followed by lumbar and cervical regions, likely due to the rich venous anatomy in the thoracic region [8,16,17]. The absence of bony involvement limits the usefulness of plain films. PESL appears isointense on T1-weighted images and is iso- to hyperintense on T2-weighted MRI with marked contrast enhancement [17,18]. MRI can clearly delineate the tumor as well as the involvement of the surrounding tissues. To complete the diagnostic workup of PESL, one should also include a contrast MRI of the entire neuraxis, body CT scan, bone marrow biopsy, bone scan, and CSF analysis [9,19].

Lymphocyte-depleted Hodgkin’s lymphoma (LDHL) is rarest subtype of classic Hodgkin’s lymphoma (CHL), initially recognized in 1966 [20], and comprising approximately < 1% of all cases of CHL [21]. Morphologically, these tumors can mimick other anaplastic malignancies including non-Hodgkin’s lymphomas, nodular sclerosis Hodgkin’s lymphoma with lymphocyte depletion, carcinoma, sarcoma, and melanoma [22]. Many earlier cases of LDHL have since been reclassified as anaplastic variants of diffuse large B-cell lymphoma or anaplastic large cell lymphoma [22]. LDHL has a male predominance (60-75% of cases) with an median age range of 30-37 years [23,24] with a predilection for nodes in the retroperitoneum, abdomen, thorax, and abdomen, with relative sparing of the peripheral lymph nodes [21,25]. A recent study has found an association between LDHL and HIV-positive status [26].

Morphologically, LDHL can be distinguished from other subtypes of CHL by the presence of numerous Hodgkin and Reed-Sternberg (RS) cells and a relative paucity of background lymphocytes, with a variable fibrotic reaction [22]. An extensive battery of immunostaining must be performed to further distinguish LDHL from diffuse B-cell lymphomas, anaplastic large-cell lymphoma, or nodular sclerosis CHL with lymphocyte depletion. A review of seven cases of LDHL showed expression for CD30, CD15, fascin, weak PAX5 and MUM-1 markers but lacked CD45, Alk-1, EMA, CD3, CD68, Mart-1 and cytokeratin [22]. For ease of reference, we have included a table outlining immunohistochemical markers for both LDHL and Non-Hodgkin’s lymphoma (NHL; Table 2).

**Table 2:** Immunohistochemical markers for Lymphocyte-depleted Hodgkin’s Lymphoma and Non-Hodgkin’s Lymphoma

**Lymphoma Subtype**	**Expressed Markers**
**LDHL**	PAX5 *(weak)*, CD30, CD15, MUM-1, fascsin, Oct.2 *or* Bob-1 (not co- expressed)
**NHL**	PAX5 *(strong)*, CD20, CD79a (B cell NHL), EMA, ALK-1, Oct.2 *and* Bob-1 (always co-expressed)

Goals of treatment echo those of any lesion causing epidural spinal cord compression: decompression of the neural elements, tissue diagnosis, and spinal stabilization [27]. In cases of primary disease presenting with spinal cord compression, surgical debulking is mandated and also allows for adequate tissue sampling for diagnosis [19]. This is followed by radiation therapy alone or in conjunction with chemotherapy but rarely with chemotherapy alone [19,28,29]. Rathmell *et al* reported a 33% actuarial survival rate in patients treated with radiation alone compared to 86% with combination therapy [30], while Monnard *et al* found that combined treatment was statistically superior to radiotherapy alone [19]. This is similar to treatment for NHL, although therapy for NHL varies according to the histological type and disease grading, with chemotherapy alone for nonindolent variants, while radiotherapy alone is used in indolent stage I and contiguous stage II NHL. This is in contrast to emerging treatment of indolent noncontiguous stage II, III, and IV NHL with antibodies directed to presenting B-cell antigens in combination with systemic chemotherapy. Aggressive NHL is treated using a combination of chemotherapy and involved-field radiation therapy, which has shown to be more effective than just chemotherapy alone. To the best of our knowledge, there have been no previous reports of a primary epidural spinal LDHL causing spinal cord compression. We herein report our findings to highlight this extremely rare subtype of CHL and also to emphasize the importance of diligent investigation in a patient that presents with back pain and unremarkable plain film xrays.

## Case presentation

### History

An 88 year-old Caucasian female was seen by the neurosurgical service with worsening pain between her scapula and difficulties ambulating. She had had upper back pain for several months. She underwent work up with plain films which did not show any abnormalities, and had been managed conservatively by pain management clinics. Over the past 4 weeks, she had developed progressive lower extremity weakness, requiring a walker for ambulation. She was admitted to hospital for medical management of her back pain and over the course of the next 48 hours, she had subjective worsening of her lower limb weakness. A review of systems was negative for malignancy or B-type symptoms.

### Examination

Physical examination was negative for lymphadenopathy. Neurologic examination was significant for a positive Rhomberg’s sign and decreased pinprick sensation in both legs extending to the hip (approximately to the L2 distribution). She had loss of proprioception in all extremities. Rectal tone was normal. Toe responses were described as extensor on the left and equivocal on the right. Blood panel showed normal values apart from slight anaemia and mildly elevated platelets.

### Imaging

**Figure 1:**
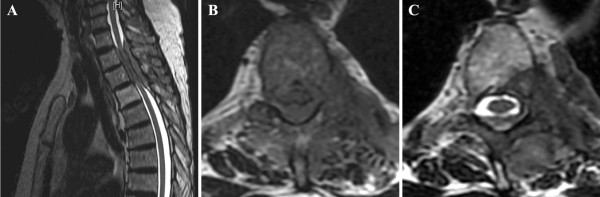
**Imaging of the spine demonstrating the epidural tumor mass *.* A)** Sagittal T2-weighted MRI showing the dorsal epidural lesion with cord compression. **B)** Axial T2-weighted MRI showing circumferential epidural compression of the spinal cord by the tumor mass with, **C)** tumor extension through the intervertebral foramen with lateral extension into the paraspinal tissues.

Since plain films had been normal, an MRI of the thoracic and lumbar spine was ordered, showing an epidural lesion from T2 to T4 levels (Figure 1A) with involvement of the posterior left T4 vertebral body (Figure 1B), posterior elements of T2 and T3, as well as extension through the left T2-3 and T3-4 neural foramina (Figures 1C & D). The lesion caused significant cord compression with evidence of mild cord edema.

### Management

The patient was taken urgently to the operating room for a decompressive laminectomy from T1-T4. The epidural mass was diligently resected to decompress the cord. Frozen tissue histology confirmed a malignant infiltrative tumor initially suggestive of a carcinoma, which prompted us to also excise the surrounding involved paraspinal tissues. Given her advanced age, extensive tumor burden, and preexisting comorbidities, we opted for a wide decompression of the spinal cord but no further aggressive surgical measures.

### Pathology

**Figure 2:**
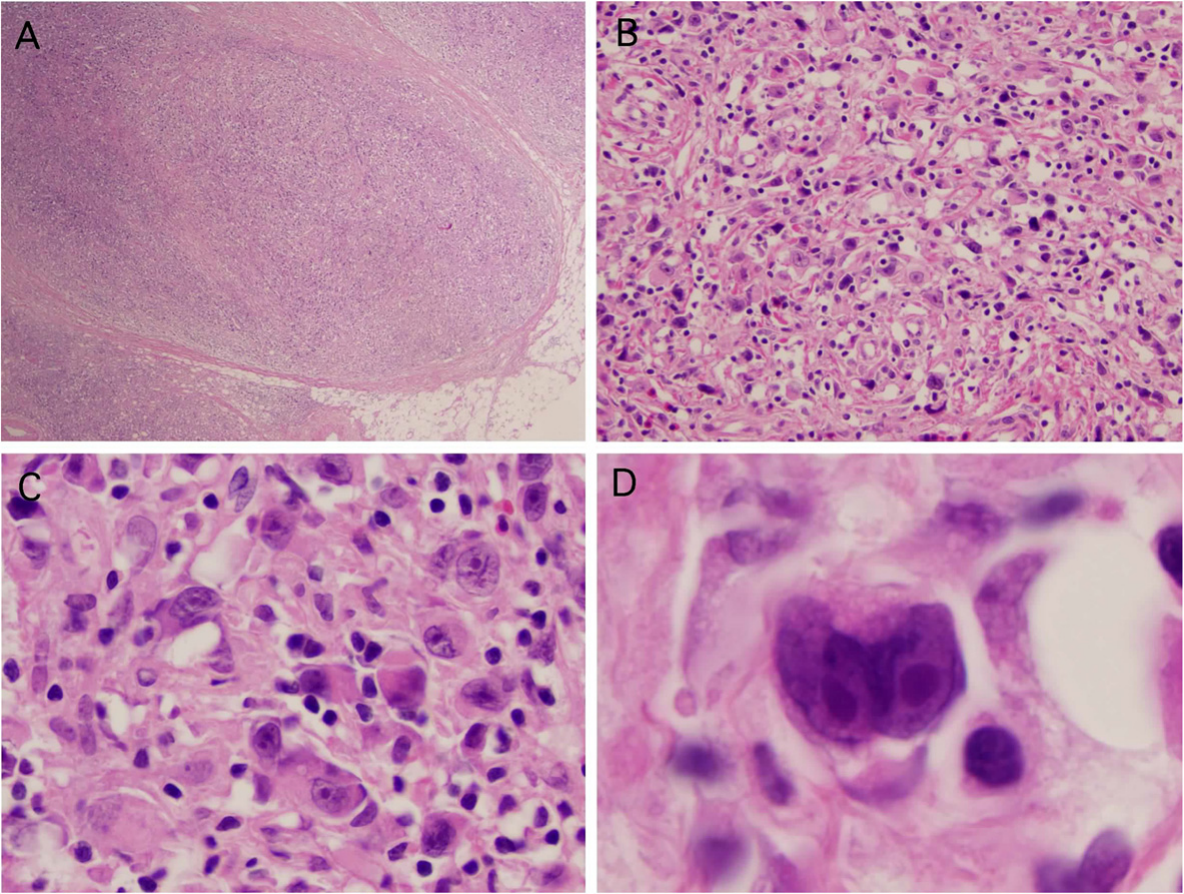
**Fresh frozen and permanent histological analysis of the tumor. A)** Initial fresh frozen sample obtained intraoperatively suggestive of a poorly differentiated carcinoma. **B)** H&E permanent section showing scattered small lymphocytes admixed amongst numerous large neoplastic Reed Sternberg cells. **C)** Neoplastic RS cells stained positive for CD30, a marker of lymphocyte activation. Paucity of lymphocytes admixed with a background of fibrosis suggests that this is lymphocyte-depleted subtype of HL.

The tumor displayed a fibrotic background without frank sclerosis, increased numbers of large atypical cells and a dearth of small lymphoid cells (Figures 2A & B). At high magnification large lymphoid cells with abundant eosinophilic cytoplasm predominated. Most of the large cells exhibited prominent inclusion-like eosinophilic nucleoli with perinucleolar clearing and many of the cells were multinucleated (Figures 2C & D). Large pleomorphic cells, multinucleated cells, and mummified cells were the predominant cell populations in the tumor. There was an abundance of CD30 + RS cells, but neither the B cell lineage marker CD20, nor CD45 were expressed by RS cells. The B-cell transcription factor Bob-1 was positive only in rare large neoplastic cells which were admixed against a scant background of predominantly T lymphocytes, but PAX5 was weakly positive in many of the cells a feature that is characteristic of classical HL. Based on the combined presence of a dominant population of RS cells with the immunophenotype characteristic of classical HL and apaucity of lymphocytes, our pathology colleagues, deduced that this patient presented with a classic example of an extremely rare form of classical HL denominated LDHL.

### Postoperative course

Following surgery, she had persistent loss of proprioception with an unsteady gait, which we attributed secondary to prolonged compression of the dorsal columns by the circumferential tumor mass. She demonstrated myelopathic symptoms with mild hyperreflexia to her lower extremities and Babinski reflexes on postoperative exam (which were present prior to surgical decompression). Systemic workup, including a PET study, did not show nodal disease or presence of extranodal involvement outside of the symptomatic thoracic region. CSF was not examined due to the primary epidural nature of the disease. Combination therapy was offered to the patient, but after extensive counseling, she opted for radiotherapy alone, not wanting to experience the possibly significant side-effects of chemotherapy and the resultant need for prolonged hospital stay to receive treatment. She thus received ten treatments of external beam radiation at 3 Gy fractions for a total of 30 Gy, from which she was experiencing mild fatigue, esophagitis, and moderate erythema to the treatment area. At the end of therapy, she had 5/5 strength in the upper and regained 4/5 strength in the lower extremities on exam. She was transferred to a palliative care unit where she lived well until she died of cardiopulmonary complications at 5 months.

## Conclusion

Bone involvement is common during the course of both Hodgkin’s and non-Hodgkin’s lymphoma, but rarely presents with epidural spinal cord compression. The initial manifestation of such lymphoproliferative disorders in the spine is a very rare entity and even more so when encountered as an isolated disease focus, hence its place on the list of differential diagnosis in patients who present with back pain and compressive myelopathy are unfamiliar to most clinicians. The clinical, radiological, and histological features of this disease can mimic other medical conditions leading to canal compromise, including pathological compression fractures, infectious lesions (such as tuberculosis and osteomyelitis), or carcinomatous deposits. This makes the correct diagnosis difficult and often leads to significant delays until effective treatment is initiated.

## Consent

Written informed consent was obtained from the patient for publication of this case report and any accompanying images. A copy of the written consent is available for review by the Editor-in-Chief of this journal.

## Abbreviations

CHL = Classic Hodgkin’s lymphoma; HL = Hodgkin’s lymphoma; LDHL =Lymphocyte-depleted Hodgkin’s lymphoma NHL = Non-Hodgkin’s lymphoma; PESL = Primary epidural spinal lymphoma RS = Reed-Sternberg.

## Competing interests

The authors have no financial or non-financial competing interests to declare.

## Authors’ contributions

EMK and GAP were responsible for the conception, design of this study, and drafting of the manuscript. FCL was responsible for data analysis, interpretation, and drafting of the manuscript. MML and POZ were responsible for data acquisition. All authors read and approved the final manuscript.

## Pre-publication history

The pre-publication history for this paper can be accessed here:

http://www.biomedcentral.com/1471-2377/12/64/prepub
